# Ultraviolet irradiation doses for coronavirus inactivation – review and analysis of coronavirus photoinactivation studies

**DOI:** 10.3205/dgkh000343

**Published:** 2020-05-14

**Authors:** Martin Heßling, Katharina Hönes, Petra Vatter, Christian Lingenfelder

**Affiliations:** 1 Institute of Medical Engineering and Mechatronics, Ulm University of Applied Sciences, Ulm, Germany; 2 Pharmpur GmbH, Königsbrunn, Germany

**Keywords:** coronavirus, SARS-CoV, SARS-CoV-2, MERS-CoV, ultraviolet, UVC, irradiation, inactivation, disinfection, Covid-19

## Abstract

**Background:** To slow the increasing global spread of the SARS-CoV-2 virus, appropriate disinfection techniques are required. Ultraviolet radiation (UV) has a well-known antiviral effect, but measurements on the radiation dose necessary to inactivate SARS-CoV-2 have not been published so far.

**Methods:** Coronavirus inactivation experiments with ultraviolet light performed in the past were evaluated to determine the UV radiation dose required for a 90% virus reduction. This analysis is based on the fact that all coronaviruses have a similar structure and similar RNA strand length.

**Results:** The available data reveals large variations, which are apparently not caused by the coronaviruses but by the experimental conditions selected. If these are excluded as far as possible, it appears that coronaviruses are very UV sensitive. The upper limit determined for the log-reduction dose (90% reduction) is approximately 10.6 mJ/cm^2^ (median), while the true value is probably only 3.7 mJ/cm^2^ (median).

**Conclusion:** Since coronaviruses do not differ structurally to any great exent, the SARS-CoV-2 virus – as well as possible future mutations – will very likely be highly UV sensitive, so that common UV disinfection procedures will inactivate the new SARS-CoV-2 virus without any further modification.

## Zusammenfassung

**Hintergrund:** Um die weltweite Ausbreitung des SARS-CoV-2 Virus zu verlangsamen, werden geeignete Desinfektionstechniken benötigt. Ultraviolette Strahlung (UV) hat bekanntlich eine antivirale Wirkung, aber Messungen zu benötigten Bestrahlungsdosen für die Inaktivierung von SARS-CoV-2 sind bisher nicht veröffentlicht worden.

**Material und Methoden:** Coronavirusinaktivierungsexperimente, die in der Vergangenheit durchgeführt wurden, werden herangezogen, um die UV-Bestrahlungsdosis für eine 90%ige Virusreduktion zu ermitteln. Die durchgeführte Analyse nutzt dabei die Tatsache, dass alle Coronaviren eine ähnliche Struktur und eine vergleichbare RNA-Länge aufweisen.

**Ergebnisse:** Die verfügbaren Daten weisen große Variationen auf, die durch unterschiedliche experimentelle Bedingungen zu erklären sind. Wenn extremere Versuchsbedingungen ausgeschlossen werden, zeigt sich, dass Coronaviren sehr UV-empfindlich sind. Der ermittelte obere Grenzwert für die log-Reduktionsdosis (90% Reduktion) beträgt ungefähr 10.6 mJ/cm^2^ (Median), während der wahre Wert vermutlich nur etwa 3.7 mJ/cm^2^ (Median) beträgt.

**Schlussfolgerung:** Da sich Coronaviren in ihrer Struktur nicht stark unterscheiden, wird auch das neue SARS-CoV-2 Virus – und mögliche zukünftige Mutationen – sehr UV-empfindlich sein, vermutlich sogar so UV-empfindlich, dass etablierte UV-Desinfektionsverfahren das neue SARS-CoV-2 Virus ohne zusätzliche Modifikationen effizient inaktivieren können.

## Introduction

The newest coronavirus disease COVID-19 is a highly transmittable and pathogenic viral infection caused by the severe acute respiratory syndrome coronavirus 2 (SARS-CoV-2), which emerged in Wuhan, China and spread around the world. So far, more than 3.8 million infections have led to more than 265,000 fatalities worldwide [1].

The responsible pathogen, SARS-CoV-2, is an enveloped single-stranded RNA virus and member of the family coronavidae of the order nidovirales. All viruses of this family exhibit very similar features. They have a spheroid shape with a diameter of about 100–150 nm, are covered with spike proteins on the outside, and have an RNA strand length of 27–32 kb on the inside [2]. Four of these coronaviruses are known to be the causative agents of common colds (HCoV-229E, HCoV-NL63, HCoV-OC43 and HCoV-HKU1), which usually only result in milder infections, but the coronaviruses MERS-CoV (MERS: Middle East Respiratory Syndrome) SARS-CoV and SARS-CoV-2 have claimed many lives [3].

To stop the spread of SARS-CoV-2, several measures such as containment, social distancing and wearing face masks have been taken. Among other steps, hygiene procedures have been intensified. The employment of liquid disinfectants is one procedure that has been successful against the 6 older coronaviruses [4]. Thermal disinfection has also been proven to be quite effective, even at temperatures as low as 60°C to 80°C [4].

Radiation disinfection, especially ultraviolet (UV) radiation, is another well-known inactivation approach for all known microorganisms and viruses that offers some advantages over liquid disinfectants and heat sterilization. It can be performed automatically and employed to disinfect surfaces, liquids, air and rooms, and it is also very energy-efficient.

**Figure 1:**
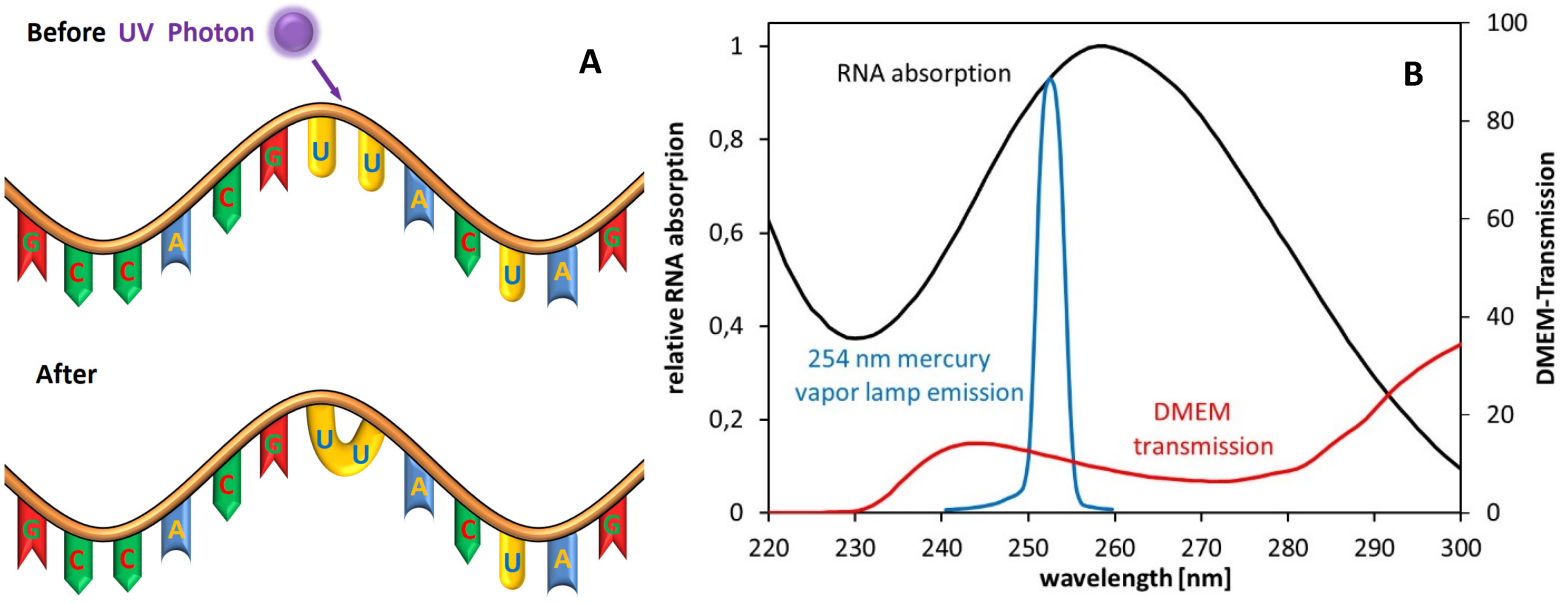
A) Scheme of UV-RNA-damaging mechanism by dimer formation. B) Relative absorption spectra of RNA, relative emission spectrum of a low-pressure mercury vapor lamp and transmission of a typical (Eagle) cell culture medium.

The ultraviolet spectrum is divided in 4 sections: Radiation with a wavelength between 100 and 200 nm is called vacuum ultraviolet radiation (VUV). It is usually not applied for disinfection purposes because it is strongly absorbed by air. The better-known ultraviolet ranges are UVC, UVB, and UVA with spectral ranges of 200–280 nm, 280–315 nm, and 315–380 nm, respectively. Among these last three UV ranges, UVC is the one with the strongest antimicrobial/antiviral properties [5], [6]. For RNA viruses, the main inactivation mechanism is illustrated in Figure 1A (Fig. 1). UV radiation is absorbed by the RNA, which leads to the formation of pyrimidine dimers, e.g., uracil dimers [6], [7].

The most common UVC light sources for many decades now have been mercury discharge lamps, especially low-pressure mercury vapor lamps, with a strong emission peak at 254 nm, which is near the RNA absorption maximum at about 260 nm, as depicted in Figure 1B (Fig. 1).

Although it is known that this kind of UVC radiation has an inactivating effect on all microorganisms and viruses, all pathogens require different UVC irradiation doses for successful inactivation. For instance, the rotavirus requires about 25 mJ/cm^2^ of 254 nm UVC radiation from a mercury discharge lamp for a 3-log reduction, but for adenovirus (Type 40), the value is approximately 6 times higher (140 mJ/cm^2^) [5], [8].

To answer the important question regarding SARS-CoV-2 and other coronaviruses as to which irradiation doses are needed for inactivation, the existing coronavirus photoinactivation results of the last 60 years have been reviewed and analyzed in this study.

## Materials and methods

Google Scholar and Pubmed were searched for different combinations of the following terms: coronavirus, inactivation, photoinactivation, disinfection, ultraviolet, radiation, and light. In addition, a number of individual viruses that belong to the families of coronaviruses although the term “coronavirus” does not occur in their name were also searched for, such as porcine epidemic diarrhea virus, transmissible gastroenteritis virus, feline infectious peritonitis virus, mouse hepatitis virus, sialodacryoadenitis virus, hemagglutinating encephalomyelitis virus, and infectious bronchitis virus.

The retrieved sources were evaluated according to the type of sample irradiated (aerosol, surface, or liquid), the type of light source (including emission peak wavelength), the inactivation effect achieved, and the applied irradiation dose.

If information on disinfection results for different irradiation doses could be found in a single article, those describing a virus reduction by approx. 3–4 log levels were selected. Results that were only displayed as illustrations without the corresponding values in the text or tables were read from previously enlarged figures.

If the necessary information was incomplete, e.g., because of missing irradiation details, the irradiation was estimated by available lamp information, provided that lamp type and distance were given or by other available means. Publications in which radiation was combined with photosensitizers or other chemical or biochemical agents were excluded.

Subsequently, these data were employed to calculate the log-reduction dose, i.e., the irradiation dose required for a 90% virus reduction.

## Results and discussion

About 30 publications of scientific investigations regarding photoinactivation of coronaviruses were identified. This included studies on CoV, SARS-CoV or MERS-CoV. An overview of the results is presented by sample condition and wavelength in Table 1 (Tab. 1).

Almost all experiments were performed with mercury vapor lamps, with a peak emission at 254 nm (UVC), which is near the RNA absorption peak in Figure 1 (Fig. 1). Individual investigations were performed with peak wavelengths at 222 nm (UVC), or 365 nm (UVA), or even with daylight.

In most studies, the authors did not intend to investigate the log-reduction doses of coronaviruses, but rather virus inactivation in various applications. In all experiments and for all coronaviruses, a successful virus inactivation was observed. However, because of the different objectives of the studies, the experimental conditions to determine the specific log-reduction doses were often difficult to identify. In many cases, information important for the present study’s analysis was missing.

To evaluate photoinactivation results, the basic inputs were the virus reduction and the applied irradiation dose. Not all authors provided the applied irradiation dose, but at least for some studies this value could be calculated as the product of irradiation duration and irradiation intensity, or it could be estimated. For some investigations, it was impossible to quantify the disinfection success exactly; in these cases, the values were estimated based on the information given in Table 1 (Tab. 1). However, for some studies, it was even impossible to roughly estimate the log-reduction dose.

The calculated and estimated results, given as log-reduction doses, exhibit extreme variability, even within the 254 nm results, ranging from 0.6 mJ/cm^2^ (bovine corona virus [9]) to 11,754 mJ/cm^2^ (SARS (CoV Urbani) [10]). Even the differences between the SARS-CoV strains were above two orders of magnitude concerning the necessary dose.

Possible reasons for this observation might be biological and biochemical differences between the coronavirus strains. However, comparing the experimental details, two other potential dominant factors attract attention:

1. The necessary irradiation doses are lower for viruses on surfaces, aerosols and pure salt solutions. When irradiation experiments were performed with the virus in different solutions, it is important to bear in mind that the solutions contain organic materials, e.g., blood products or residue from cell culture medium. These solutions exhibit very high absorption of the applied UVC radiation, resulting in much lower irradiances for viruses that are deeper inside the sample. This effect is clearly illustrated in Figure 1B (Fig. 1) (culture medium transmission) and by the results of Terpstra et al. [11]. Those authors were aware of the absorption of their samples and presented results with 10%- and 30%-virus-containing plasma within the irradiated samples. Although it was the same coronavirus (transmissible gastroenteritis virus (TGEV)) and the same experimental setup, the results differ by a factor of 3.1.
  Most authors did not measure the UVC absorption properties of their biological materials because it was of no importance for their research task; thus, it is almost impossible to extract the role of the absorption in the calculation of the necessary irradiation doses for a 90% virus reduction. In consequence, the lower values for the log-reduction doses, mostly from viruses in salt solutions, surfaces or aerosols, might be a more realistic approach to determine the true virus log-reduction dose.

1. Several investigators performed their irradiation experiments in microtiter plates (MTPs). This might become a problem if the plates are too close to the irradiation source, e.g., in the range of only a few centimeters. Besides the risk of heating the sample and subsequently increased sample evaporation, determination of the irradiation intensity inside the MTP wells becomes difficult. The MTP well walls shade the virus-containing samples from irradiation that does not originate directly above the well. Hence, the true irradiation intensity within a well is probably quite low compared to the intensity measured by a photodetector at the same distance.

Table 1 (Tab. 1) states whether a study exhibited one or two of these complications for the intended log-reduction determination. Actually, all calculated extreme values in Table 1 (Tab. 1) seem to be influenced by both complicating factors, and therefore these 254 nm values [12], [13], [10] were omitted in the further analysis. The data of the Berne torovirus [14] were also excluded, because of structural differences between toroviruses and coronaviruses [15].

All coronaviruses exhibit a similar structure and a single-stranded RNA length of about 30 kb, allowing the conclusion that they also feature very similar UVC absorption and UVC disinfection properties. It is therefore justified to consider all coronaviruses alike in terms of the investigated UVC-based log-reduction and to summarize the individual results.

This leads to a total UVC median log-reduction dose of 10.6 mJ/cm^2^ (average 11.9±11.4 mJ/cm^2^) These values were calculated without the torovirus data and outliers, but the input included viruses in media that probably had higher UVC absorption, leading to reduced photoinactivation. Therefore, this 10.6 mJ/cm^2^ is probably not the real value for the log-reduction dose, but instead could be considered as an upper limit.

Recalculation of the UVC log-reduction dose without using results from higher-absorption media should lead to more realistic values. In this case, the total median log-reduction dose would be 3.7 mJ/cm^2^ (average 5.8±5.5 mJ/cm^2^).

This overview covers all coronaviruses and both UVC wavelengths (222 nm and 254 nm), but without results obtained from studies with probably high absorption media. The obtained results agree well with UVC inactivation data for other ssRNA viruses, such as influzenza A with log-reduction doses around 2 mJ/cm^2^ [5] or the ssRNA bacteriophage MS2 with log-reduction doses of about 20 mJ/cm^2^ [5].

So far, most instances of successful coronavirus inactivation have been performed using mercury vapor lamps with peak emission at 254 nm. To reduce the use of the toxic mercury, it seems possible that these vapor lamps will be replaced in the future by 222-nm excimer lamps or by 270-nm LED. Since the RNA absorption strengths are similar, the disinfecting effect at these wavelengths will probably be approximately the same as with mercury vapor lamps. However, this should be investigated in more detail in the future, since absorptions in the lipid envelope might have a larger influence on virus inactivation than currently assumed.

Because there are only single results available for the effect of 365 nm (UVA) and daylight, the focus of this analysis is on UVC coranavirus inactivation. Both irradiation methods demonstrated a virus reduction, albeit seemingly much less effective than that achieved with 254 nm irradiation. Nevertheless, these longer wavelengths might also be of future interest because their absorption in samples with organic materials is much lower, resulting in higher penetration depths which may allow virus inactivation of larger volumes.

## Conclusion

To date, UVC radiation has been effective against all coronaviruses in all published investigations, although the absorption properties of the sample media reduced inactivation success. The calculated upper limit for the log-reduction median dose (in low-absorbance media) is 10.6 mJ/cm^2^, but the probably more precise estimation is 3.7 mJ/cm^2^.

These results were obtained by investigations on many different coronaviruses, including SARS-CoV and MERS-CoV, but not SARS-CoV-2. Nevertheless, it can be assumed that they are also applicable for SARS-CoV-2 and all future mutations. RNA mutations might have a strong influence on the pathogenicity of a virus, but they do not result in larger structural differences, especially concerning the UV absorption properties of the RNA, which are the main cause for the antiviral effect of ultraviolet radiation.

The above-mentioned log-reduction doses are in the same order of magnitude or even smaller than log-reduction doses for other important pathogens, such as *Staphylococcus aureus*, *Escherichia coli*, *Klebsiella pneumonia* or *Candida albicans* [5]. They are also low compared to UVC irradiation recommendations, for instance, the international standard for UV disinfection of drinking water [16] with its recommended irradiation dose of 40 mJ/cm^2^. Therefore, it can be concluded that existing UVC disinfection systems and procedures will be sufficient to deal with all coronaviruses, including SARS-CoV-2.

## Notes

### Competing interests

The authors declare that they have no competing interests.

## Figures and Tables

**Table 1:**
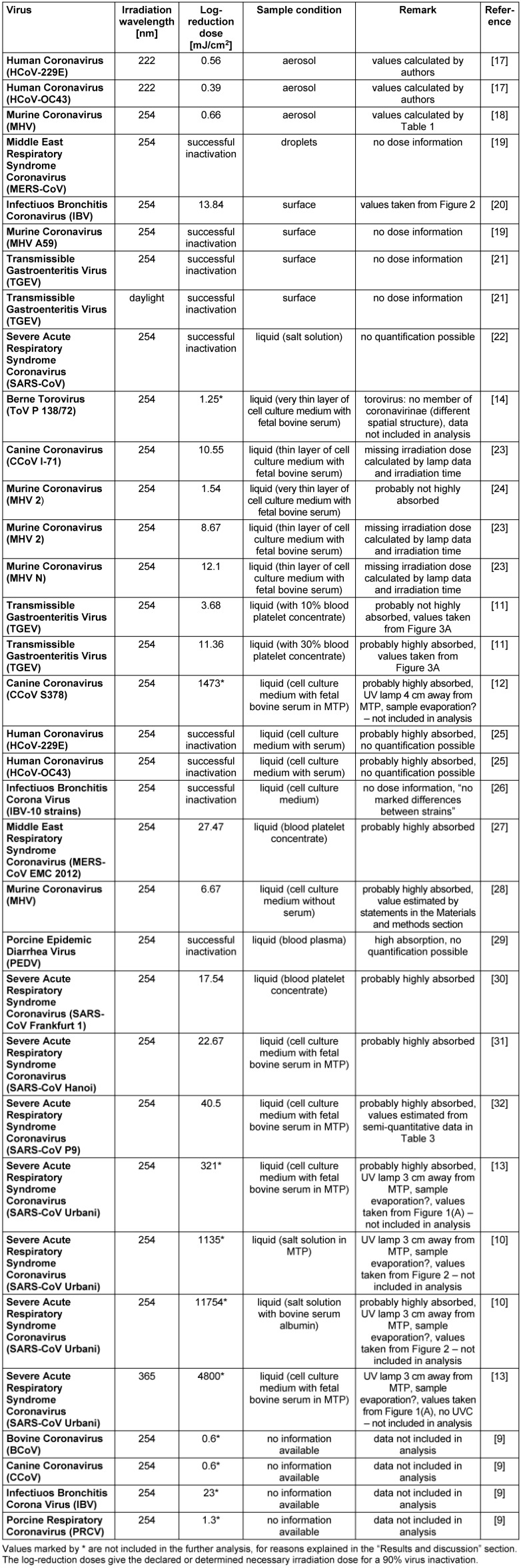
Overview of published and analyzed coronavirus photoinactivation investigations sorted by sample condition
